# Four translation initiation pathways employed by the leaderless mRNA in eukaryotes

**DOI:** 10.1038/srep37905

**Published:** 2016-11-28

**Authors:** Kseniya A. Akulich, Dmitry E. Andreev, Ilya M. Terenin, Victoria V. Smirnova, Aleksandra S. Anisimova, Desislava S. Makeeva, Valentina I. Arkhipova, Elena A. Stolboushkina, Maria B. Garber, Maria M. Prokofjeva, Pavel V. Spirin, Vladimir S. Prassolov, Ivan N. Shatsky, Sergey E. Dmitriev

**Affiliations:** 1 Engelhardt Institute of Molecular Biology, Russian Academy of Sciences, Moscow, 119991, Russia; 2 School of Bioengineering and Bioinformatics, Lomonosov Moscow State University, Moscow, 119234, Russia; 3 Belozersky Institute of Physico-Chemical Biology, Lomonosov Moscow State University, Moscow, 119234, Russia; 4 Institute of Protein Research, Russian Academy of Sciences, Pushchino, Moscow Region, 142290, Russia; 5 Department of Biochemistry, Biological Faculty, Lomonosov Moscow State University, Moscow, 119991, Russia

## Abstract

mRNAs lacking 5′ untranslated regions (leaderless mRNAs) are molecular relics of an ancient translation initiation pathway. Nevertheless, they still represent a significant portion of transcriptome in some taxons, including a number of eukaryotic species. In bacteria and archaea, the leaderless mRNAs can bind non-dissociated 70 S ribosomes and initiate translation without protein initiation factors involved. Here we use the Fleeting mRNA Transfection technique (FLERT) to show that translation of a leaderless reporter mRNA is resistant to conditions when eIF2 and eIF4F, two key eukaryotic translation initiation factors, are inactivated in mammalian cells. We report an unconventional translation initiation pathway utilized by the leaderless mRNA *in vitro*, in addition to the previously described 80S-, eIF2-, or eIF2D-mediated modes. This mechanism is a bacterial-like eIF5B/IF2-assisted initiation that has only been reported for hepatitis C virus-like internal ribosome entry sites (IRESs). Therefore, the leaderless mRNA is able to take any of four different translation initiation pathways in eukaryotes.

Translational properties of both prokaryotic and eukaryotic mRNAs are largely dictated by their 5′ untranslated regions (5′ UTRs). However, a fraction of mRNA transcripts with either a very short 5′ UTR or even completely lacking it (i.e., leaderless) occurs naturally in the living world. Leaderless mRNAs are especially common in Archaea^1^ and represent the only mRNA type in mammalian mitochondria^2^. They are also abundant in a variety of bacteria species^3^. In eukaryotes, nuclear-encoded leaderless transcripts are widely represented across a number of primitive unicellular organisms^4^,^5^. Thus, this peculiar class of mRNAs is present in all three domains of life.

Although these molecules lack any special nucleotide sequences at their 5′-termini, except for the AUG itself, they can efficiently direct protein synthesis in bacterial, archaeal, or mitochondria systems *in vitro* as well as *in vivo*^6^,^7^,^8^,^9^. The structural peculiarity of the leaderless mRNA imparts some unusual translational properties. The ability of the lambda phage leaderless *cI* mRNA to bind directly to non-dissociated 70 S ribosomes in the presence of fMet-tRNA^Met^_f_ was initially reported for bacterial systems^10^. This binding did not require any additional protein factors. Authenticity of this unconventional translation initiation mechanism was thoroughly demonstrated later by multiple experiments both *in vitro* and *in vivo*^6^,^11^,^12^, including an elegant approach when ribosomal subunits were cross-linked to prevent their dissociation^11^. It has been recently shown that the 70 S mediated translation initiation can be utilized also by 5′ distal cistrons in polycistronic bacterial messengers^13^ and thus seems to be not exclusive for the leaderless mRNAs. However, in the case of 5′ proximal cistrons it definitely represents a specific pathway for this particular mRNA type^10^,^11^.

An alternative mechanism for the leaderless mRNA translation initiation in bacteria and archaea is a Met-tRNA^Met^_f_-assisted 30 S recruitment that is promoted by initiation factor IF2^7^,^10^,^14^, an ortholog of the eukaryotic eIF5B^15^. IF2 is known to stabilize both the initiator tRNA and mRNA binding to the bacterial ribosome^16^,^17^. According to kinetic studies, IF2 and Met-tRNA^Met^_f_ association usually precedes mRNA binding to the 30 S subunit, although the precise order is thought to depend on a particular mRNA species^16^. In the case of the leaderless mRNA, the initial IF2 binding is mandatory for the 30S-mediated pathway, and the elevated IF2 level selectively stimulates its translation both *in vitro* and *in vivo*^7^. Stimulation of the leaderless mRNA translation by IF2 orthologs is also revealed for archaeal and mitochondrial systems^9^,^18^.

In eukaryotes, a short leader is known to impair the fidelity of translation initiation^19^. Nevertheless, leaderless mRNAs are shown to be preferentially translated in trophoblasts of *Giardia lamblia*^20^ and can be effectively expressed in yeast^21^. The ability of the eukaryotic ribosomes to translate a leaderless transcript was also confirmed for rabbit reticulocyte lysate (RRL), the most widely used mammalian cell-free translation system^7^,^22^,^23^. This system, however, is known to poorly reconstitute conditions in the living mammalian cells and is prone to certain artifacts (for review, see ref. 24). Moreover, translational properties of the leaderless mRNA have never been studied in living cells of a higher eukaryote.

Previously, using a mammalian translation reconstituted system, we showed that the *cI*-derived leaderless mRNA is able to directly bind eukaryotic 80 S ribosomes and in the presence of Met-tRNA_i_ can trigger translation without any initiation factor^23^. Similarly to bacteria, the leaderless mRNA in eukaryotes is not limited to this way in initiating translation, since a canonical 40S-mediated eIF2-dependent mechanism can also successfully operate on mRNAs with 5′-terminal AUG codons^23^,^25^. Yet, such 48 S initiation complex is disassembled by eIF1^23^,^25^, a protein obviously present in a living cell. Later, we showed that another 48 S complex assembly pathway, namely the eIF2D-mediated one, can be also exploited by the leaderless mRNA^26^. Thus, this transcript is able to choose at least from three distinct strategies to direct its translation *in vitro*. This versatility could provide the leaderless mRNA with peculiar translational properties *in vivo* or at least impart some resistance to various kinds of cellular stress. We previously demonstrated such translation plasticity for the hepatitis C (HCV) virus internal ribosome entry site (IRES) containing mRNA, which can utilize both canonical eIF2-dependent and unconventional eIF5B-mediated initiation complex assemblies^27^. Indeed, the HCV IRES-directed translation is highly resistant to stresses resulting in eIF2 inactivation^27^,^28^.

In this study, we show that *cI*-derived leaderless transcript encoding firefly luciferase can direct translation in living mammalian cells under conditions of severe stress when the canonical cap-dependent ribosomal scanning is strongly compromised. Furthermore, similarly to the HCV IRES, the leaderless mRNA is able to form the eIF5B-medated 48 S translation initiation complex. Consequently, this is an unprecedented example of an mRNA that can utilize any of four different translation initiation pathways in eukaryotic systems.

## Results

### Translation of the leaderless mRNA in living mammalian cells is relatively resistant to various stress conditions

Translational properties of any leaderless mRNA have never been studied in living mammalian cells before. To perform such an analysis, we prepared a firefly luciferase (Fluc) reporter construct starting with the *cI* mRNA 5′ terminal sequence (40 nt in length, Supplementary Fig. 1). This mRNA had only one nucleotide (G) before the start AUG codon. In parallel, we used two other Fluc encoding constructs that had either the human β-actin 5′ UTR or an artificial 20 nucleotide-long GA(CAA)_5_GAA leader, hereafter called (CAA)_5_cI (Supplementary Fig. 1).

**Figure 1:**
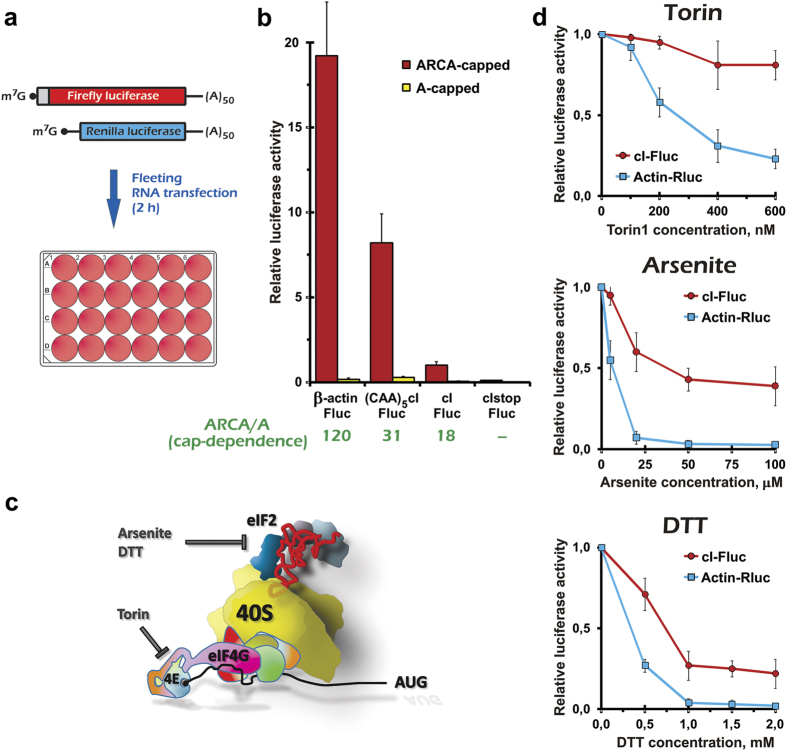
Leaderless mRNA translation is relatively resistant to translation initiation inhibitors in transfected mammalian cells. (**a**) Schematic representation of the Fleeting mRNA transfection procedure (FLERT). (**b**) Translation efficiency of the reporter mRNAs constructs. HEK293T cells were transfected with ARCA- (Anti-Reverse Cap Analog) or A-capped polyadenylated Fluc constructs having indicated 5′ leaders, along with the similarly prepared ARCA-capped Actin-Rluc mRNA. In the cIstop-Fluc construct, the 5′ terminal AUG was replaced with UAA. Fluc activity was analyzed after 2 h incubation, normalized to Rluc activity units and represented as a ratio to the value for the capped cI-Fluc mRNA. The mean absolute value for Fluc in this case was 221,805 luciferase units, Rluc – 16,238,226 units, while the background (no-transfection control) values were 60 and 150 units, respectively. Cap-dependence (*i.e*. ratio of translation efficiency of the ARCA- *vs*. A-capped mRNAs) is indicated at the bottom. (**c**) Schematic representation of inhibitors action on the translation initiation pathway. (**d**) Translation inhibition by the drugs as revealed by the FLERT technique. Luciferase activities are normalized to that in wells without the inhibitors.

To exclude any possible impact of transcription-related events on the mRNA appearance and expression, we took advantage of the mRNA transfection technique^29^. The corresponding capped polyadenylated transcripts were mixed with a similarly prepared *Renilla* luciferase (Rluc) mRNA having the human β-actin 5′ UTR. The mixture was further transfected into cultured human cells seeded onto a 24-well plate 12-16 h before the transfection (Fig. 1a). The transfection procedure was performed in a way that minimally disturbs the cell culture (see Methods section for details). After only 2 h of mRNA translation, cells were harvested and the luciferase activities were measured. This procedure, which we called FLEeting mRNA Transfection (FLERT), enabled us to minimize secondary (non-specific) effects caused by any additions of drugs or by the RNA transfection itself (Akulich *et al*., in preparation).

The leaderless mRNA translated in living cells produced a luciferase activity at a level comparable to that directed by the two leadered mRNAs (Fig. 1b). Interestingly, the 5′ terminal m^7^G cap stimulated the leaderless mRNA translation, although its cap-dependence was lower than for two other mRNAs (Fig. 1b). A similar transcript with the 5′ terminal AUG replaced by UAA produced only a background level of luciferase activity, indicating that the 5′ terminal AUG was the only translation initiation site responsible for producing the active enzyme.

We then exposed the transfected cells to various stress conditions or small molecule drugs (Fig. 1c). Firstly, we treated the cells with torin1, an mTOR kinase inhibitor^30^. Hereafter, we applied the stress inducer immediately (~5 min) prior to the transfection procedure. This treatment brought about up to 4-fold inhibition of the control (capped Actin-Rluc) mRNA translation (Fig. 1d), most likely due to inactivation of cap-binding factor eIF4F^30^. In contrast, translation of the leaderless mRNA was almost completely resistant to the inhibition of mTOR. This correlates with the lower cap-dependence of the leaderless mRNA shown in Fig. 1b.

Secondly, we induced a severe oxidative stress by addition of sodium arsenite known to induce robust eIF2α phosphorylation. The resistance of the mRNA translation to the arsenite stress is regarded as a specific marker for relaxed eIF2-dependence^27^,^31^,^32^,^33^. Arsenite at concentrations of 20 μM or more inhibited translation of the Actin-Rluc mRNA almost completely, in accordance with our previous data^27^,^31^. Under these conditions, the leaderless mRNA translation was not only partially resistant, but it also remained rather efficient, demonstrating a relative advantage over the Actin-Rluc mRNA (Fig.1d, Supplementary Fig. 2). Another test that could be used to assess a relaxed eIF2 dependency *in vivo* is translation resistance to unfolded protein stress caused by dithiotreithol^27^,^31^,^33^. Again, the leaderless mRNA translation showed pronounced resistance to this kind of stress (Fig. 1d), and a ten-fold relative advantage in the resistance over the Actin-Rluc mRNA at 2 mM of DTT was demonstrated (Supplementary Fig. 2). We conclude that the leaderless mRNA can operate partially in an eIF2-independent manner *in vivo*.

**Figure 2:**
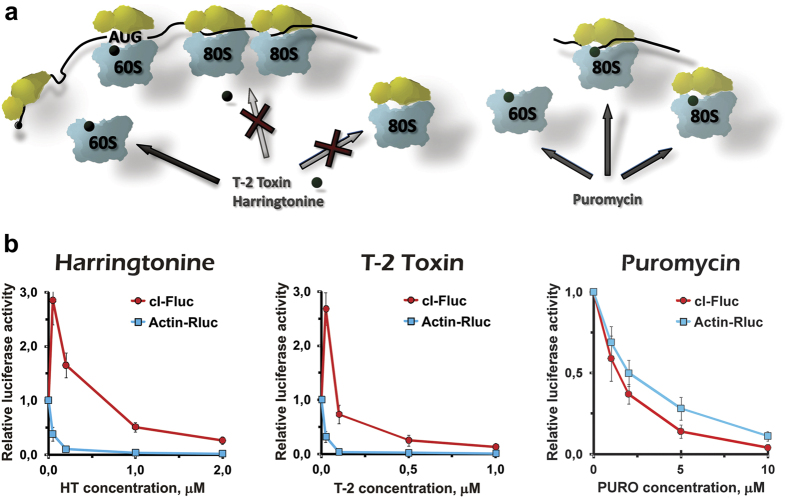
Leaderless mRNA translation is relatively resistant to selective 60 S targeting inhibitors. (**a**) Schematic representation of the inhibitors action. Note that harringtonine and T-2 toxin are unable to enter elongating ribosomes and pre-associated vacant 80 S particles, while puromycin binds the ribosomes at any stage. (**b**) Translation inhibition by the drugs as revealed by the FLERT technique. Luciferase activities are normalized to those in wells without the inhibitors.

Antibiotics that target ribosomes are also a powerful tool for studying translation mechanisms^34^,^35^. Both harringtonine and T-2 toxin block elongation by inhibiting peptidyl transferase center of the ribosome^36^. Due to their limited ability of binding elongating 80 S ribosomes within a polysome as well as vacant 80 S particles, they preferentially target 60 S subunits and thus arrest *de novo* assembled 80 S at the start of the coding region (Fig. 2a)^35^,^37^,^38^. Consequently, treating cells with these elongation inhibitors paradoxically doesn’t lead to polysomes stabilization but to their disassembly^35^,^37^. In our experiment, adding harringtonine or T-2 toxin in concentrations as few as ~0.1–0.2 μM almost completely blocked Actin-Rluc translation in the cultured cells (Fig. 2b). In contrast, translation of the leaderless mRNA was unexpectedly up-regulated at low drug concentrations and was remarkably resistant to higher doses (Fig. 2b and Supplementary Fig. 2). This partial resistance could be explained by a utilizing the non-canonical translation initiation pathway based on direct binding to the non-dissociated vacant 80 S ribosome, as was previously shown for the *cI*-derived transcripts *in vitro*^23^.

Comparatively, puromycin, a well-known aminonucleoside antibiotic that causes premature peptide chain termination^34^,^36^, inhibited the cI-Fluc mRNA translation even stronger than did for the Actin-Rluc mRNA (Fig. 2b). This result was reproduced with another Fluc/Rluc mRNA pair (data not shown). It was fairly predictable for a non-specific elongation inhibitor, since the Fluc coding region is ~1.8 times longer than the Rluc one. Similarly to harringtonine and T-2 toxin, puromycin binds the A-site of the large ribosome subunit^34^,^36^. However, contrary to the above two drugs, it enters 60 S subunit irrespectively of whether the latter is involved in the 80 S formation or not (Fig. 2a). Thus, it non-specifically abrogates polypeptide elongation at any stage and can be regarded as a negative control in our comparison of the mRNAs.

### Peculiar properties of the leaderless mRNA translation in mammalian and yeast cell-free systems

To investigate these properties further, we performed *in vitro* translation experiments in the self-made mammalian cell-free system that closely recapitulates translation in living cells^24^. The advantage of an *in vitro* system lies in the opportunity to vary concentration of the reaction components in a wide range. Under our standard conditions, we observed a ratio of the mRNA translation efficiencies similar to that obtained *in vivo* (~1:7 in favor to the 5′ UTR containing mRNA; see Supplementary Fig. 3a). Thus, we took these conditions as a starting point for further experiments.

Firstly, we analyzed effects of Mg^2+^ concentration on translation of both mRNAs. Translation of the leadered mRNA was only slightly stimulated by additional magnesium (up to +1,5 mM to that contained in the lysate) and then quickly decreased, as expected^39^. In contrast, the leaderless mRNA translation was explosively risen with increasing Mg^2+^ content and was still efficient even at non-physiologically high (+4 mM) Mg^2+^ concentration. Elevated magnesium is known to block ribosome dynamics and thus should make the ribosome less capable of binding mRNA *via* the conventional translation pathway that involves sequential acts of subunit association/dissociation^40^. Thus, these results may provide additional, albeit indirect, evidence for the capability of using a non-canonical pathway(s) by the leaderless mRNA.

**Figure 3:**
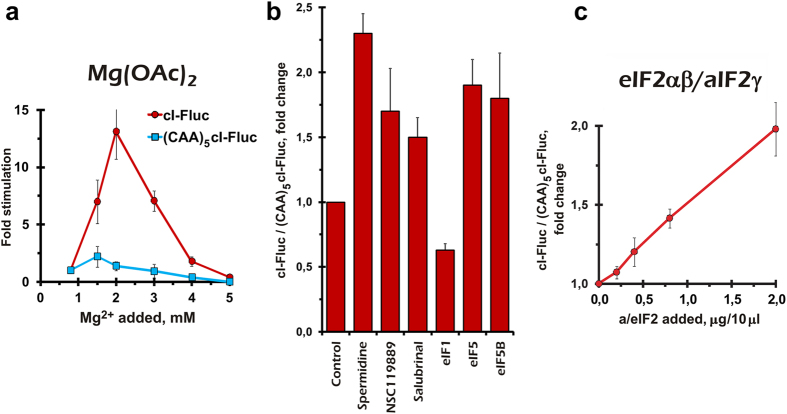
Peculiar properties of the leaderless mRNA translation in the cell-free systems. (**a**) Selective stimulation of the cI-Fluc mRNA translation by elevated magnesium concentration in cytoplasmic extract of Krebs-2 mouse ascites carcinoma cells. Note that only the concentration of Mg(OAc)_2_ in the reaction buffer is shown (without considering an endogenous Mg^2+^ concentration in the extract). The mean absolute values for cI-Fluc and (CAA)_5_cI-Fluc at 0.8 mM Mg^2+^ were 40,722 and 255,720 luciferase units, respectively. (**b**) Differential effects of the small molecule drugs and some translation initiation factors on translation of the cI-Fluc and (CAA)_5_cI-Fluc mRNAs in Krebs-2 cells extract (p < 0.05). Final concentrations of the additives were as follows: 1.5 mM spermidine-HCl; 250 μM NSC119889; 25 μM salubrinal; 10 ng/μl eIF1; 30 ng/μl eIF5; 50 ng/μl eIF5B. (**c**) Relative resistance of the leaderless mRNA translation to inhibition by recombinant eIF2αβ/aIF2γ chimeric protein in the yeast cell-free system. The luciferase activity values for the cI-Fluc mRNA were normalized to that of the (CAA)_5_cI-Fluc mRNA. The mean absolute values for cI-Fluc and (CAA)_5_cI-Fluc at the control point were 104,678 and 225,057 luciferase units, respectively. Error bars represent the standard deviations of the mean values for three independent experiments.

We then analyzed impacts of several other molecules known to affect translation initiation (Fig. 3b). Spermidine, a polyamine that lowers ribosome subunit exchange rate and at elevated concentrations stabilizes monosome particles^41^, also produced a differential effect on translation of the two reporter mRNAs. We observed similar effects with NCS119889 and salubrinal, two drugs that interfere with eIF2 function^28^,^42^. Addition of some translation initiation factors also differentially affected the reporter mRNAs translation. eIF1 predictably inhibited cI-Fluc translation to a slightly larger extent than the leadered one, in accordance with its role in destabilization of the canonical 48 S complex formation at the 5′-terminal AUG^23^,^25^. By contrast, eIF5 gave a relative advantage to the leaderless mRNA (Fig. 3b), probably by stimulating the 5′-terminal AUG recognition and blocking ribosomal sliding^43^,^44^. Interestingly, eIF5B, the eukaryotic homolog of bacterial IF2, also differentially affected the reporter mRNAs translation (Fig. 3b). This could be explained either by stabilization of the 48 S complex and inhibition of its sliding^44^,^45^ or by stimulation of an alternative translation initiation pathway in a manner similar to bacterial, archaeal, and mitochondrial IF2^46^; see below.

As we have shown previously, aIF2, an archaeal homolog of eIF2, can be used to investigate translation initiation mechanisms in eukaryotic systems^47^. aIF2 is able to substitute for eIF2 in 48 S initiation complex formation, but, since aIF2γ has distinct requirements for GTP hydrolysis, the 48 S cannot be processed further to the 80 S. Consequently, aIF2 outcompetes for eIF2 and inhibits eIF2-dependent translation^47^. In this work, we took advantage of this approach by utilizing a recombinant e/aIF2 chimera consisting of yeast eIF2α and eIF2β subunits combined with archaeal aIFγ (Arkhipova *et al*., in preparation). Since the yeast eIF2 subunits could poorly match the mammalian system, for this test we prepared a cytoplasmic extract from *S. cerevisiae* cells (Supplementary Fig. 3b). As it was expected, the chimeric eIF2αβ/aIF2γ protein inhibited translation of both cI-Fluc and (CAA)_5_cI-Fluc mRNAs but to a different extent (Supplementary Fig. 3c), leading to a relative advantage of the leaderless mRNA translation (Fig. 3c). This observation may additionally indicate the ability of this mRNA to use an alternative translation initiation pathway in the cell-free system.

### eIF5B-dependent 48 S complex formation is a novel translation initiation pathway for the leaderless mRNA

We previously showed that the leaderless mRNA can form translation initiation complexes by both the canonical eIF2-dependent pathway and two unconventional pathways: direct 80 S binding and eIF2D-mediated 48 S formation^23^,^26^. Apart from these three modes, another mechanism of the initiator tRNA delivery and ribosome initiation complex formation has been documented for a few eukaryotic mRNA molecules, namely, the eIF2-independent eIF5B-assisted pathway^27^,^48^. Taking into account our results obtained in the cell-free translation system (Fig. 3b), we decided to check the possibility for the *cI*-derived mRNA to form the 48 S translation initiation complex *via* the “bacterial-like” eIF5B-mediated Met-tRNA_i_ binding.

**Figure 4:**
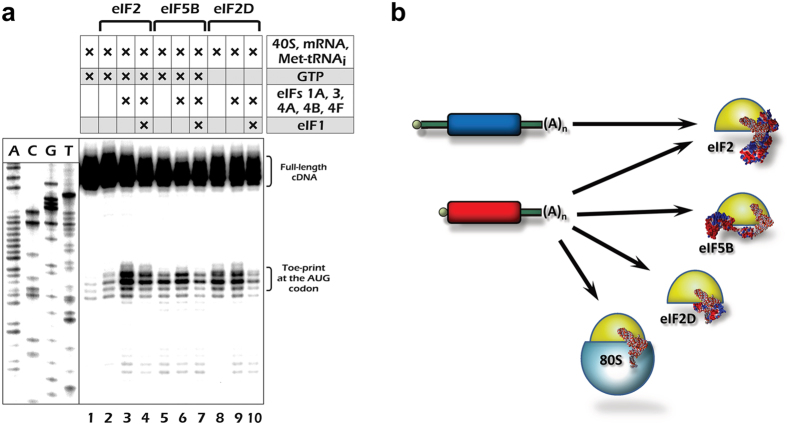
The fourth translation initiation pathway for the leaderless mRNA. (**a**) 48 S pre-initiation complex reconstitution from purified mammalian components on capped cIlacZ transcript. The assembled complexes were visualized by the toe-printing assay. Cross signs denote the components added to the reaction mixture. Sequencing lanes obtained with the same primer and the corresponding cDNA are shown on the left. (**b**) Schematic representation of four translation initiation pathways employed by the leaderless mRNA in eukaryotes.

As shown in Fig. 4a (lanes 5–7), the leaderless mRNA is indeed able to form the 48 S initiation complex in the presence of just eIF5B and Met-tRNA_i_, although the assembly is significantly stimulated by a full set of eukaryotic translation initiation factors except eIF1 (*cf*. lanes 5 and 6). In its turn, eIF1 inhibits the 48 S complex assembly (*cf*. lanes 6 and 7). A similar stimulation by eIF1A, eIF3, and eIF4s as well as inhibition by eIF1, known for the canonical eIF2-dependent 48 S complex assembly at the 5′ terminal AUG^23^,^25^, was confirmed (lanes 2–4). This is not the case for eIF2D-mediated 48 S formation^26^, which does not require eIF1A, eIF3, and eIF4s, although it was also inhibited by eIF1 (lanes 8–10). Thus, the leaderless mRNA is able to form the 48 S pre-initiation complex *via* the three distinct mechanisms, and finally (with addition of the previously described direct 80 S binding mode) it can form the 80 S complex *via* four distinct pathways.

## Discussion

Eukaryotic mRNAs are known to utilize a wide spectrum of translation initiation pathways^49^,^50^. In particular, the critical step of initiator tRNA delivery to the ribosomal complex may be served by any of four different modes, which are mediated by one of eukaryotic initiation factors: eIF2 (for review, see ref. 43), eIF2D and its homologs MCTS1•DENR^26^,^51^, eIF5B^27^,^48^, or it may even proceed without any initiation factors at all^23^. Notably, these four mechanisms have been shown previously for distinct mRNA species.

Here we describe a messenger RNA that is able to utilize any of these translation initiation modes: the leaderless mRNA (Fig. 4b). Such a diversity provides this mRNA with a flexibility that enables its translation to be highly resistant to various cell stress conditions, including those when eIF2 or eIF4F are inactivated. Thus, its translation partially escapes the control of two major eukaryotic translation regulatory pathways and resembles that of some virus IRES-containing mRNAs^52^,^53^. In this respect, it is interesting to note that translation of natural mammalian mRNAs with extremely short 5′ leaders (*e.g*. so-called TISU mRNAs) were also found to be resistant to some kind of cell stress^54^. This situation echoes the bacterial systems where leaderless mRNAs are preferentially translated under particular conditions that induce inactivation of some translation machinery components^11^,^55^,^56^. Recently, a systematic genome-wide 5′ RACE analysis by the nanoCAGE approach^57^ revealed dozens of human mRNA transcripts with 5′ leaders as short as 2 or 3 nt. This list includes mRNAs encoding such important regulators and enzymes as a diphthamide biosynthesis enzyme (DPH1), DNA polymerase epsilon 4 subunit (POLE4), AMP-activated protein kinase subunit 1 (PRKAA1), a lysosome biogenesis complex subunit (BLOC1S1), *etc*.^57^. The actual number of leaderless mRNA molecules in cells may be even higher, since many promoters produce transcripts with heterogenic 5′ termini.

Leaderless mRNAs can operate in all three domains of life^46^,^50^. Their unusual properties were well studied in bacteria and archaea, where at least two alternative initiation pathways of their translation were documented^7^,^10^,^11^,^14^. One of these mechanisms, the Met-tRNA_i_-dependent direct binding to the non-dissociated ribosome, was reproduced in eukaryotic systems before^23^. The second pathway in bacteria is the Met-tRNA^Met^_f_-assisted 30 S recruitment promoted by initiation factor IF2. In the present work, we confirm that a homologous pathway, eIF5B-mediated 48 S complex assembly, is also possible for the leaderless mRNA in eukaryotes. IF2/eIF5B is a universally conserved protein that is necessary for translation initiation in all forms of life^15^. Keeping in mind that leaderless mRNAs may be regarded as molecular fossils of protein encoding transcripts, we propose that these two pathways, the Met-tRNA-dependent 70 S/80 S ribosome binding and the IF2/eIF5B-mediated Met-tRNA delivery, were the two ancestral forms of translation initiation in all three kingdoms of life.

## Methods

### Reagents

The following reagents were used in this study: torin1 (Tocris Bioscience), harringtonine (LKT Laboratories), T-2 toxin (Cayman), spermidine trihydrochloride (Sigma-Aldrich), salubrinal and NSC119889 (Santa Cruz Biotechnology). Sodium ortho-arsenite (in the form of dihydroarsenite, NaH_2_AsO_3_) was kindly provided by Pavel Ivanov (MSU). 10 mM harringtonine, T-2 toxin, salubrinal and NSC119889 stock solutions in DMSO, 100 μM torin1 in DMSO, 1 M spermidine and arsenite water solutions were prepared and stored at −85 °C.

### Plasmid constructs and *in vitro* transcription

The plasmids pcIlacZ, pActin-Fluc, and pActin-Rluc were described earlier^23^,^24^. pcI-Fluc was prepared by insertion of the T7 promoter and the first 40 nt of the *cI* cDNA from the pcIlacZ plasmid into the pGL3 vector (for details, see Supplementary Fig. 1). It is important to note that the *cI* start codon was the only AUG among the first 100 nt and that there were no in-frame AUGs in the ORF throughout the first 29 codons of the Fluc coding region. Thus, the active enzyme could only be produced from the 5′ terminal (cI) AUG. For synthesis of the polyadenylated mRNAs encoding the firefly and *Renilla* luciferases, a 50T-tailed PCR product was used as a template, as described previously^58^. To obtain the templates for (CAA)_5_cI-Fluc and cIstop-Fluc mRNAs, the forward primers CCGTAATACGACTCACTATAGACAACAACAACAACAAGAAATGAGCACAAAAAAGAAACC and GTAATACGACTCACTATAGTAAAGCACAAAAAAGAAACC were used instead of a regular T7 promoter primer. Transcription was performed using the RiboMAX kit (Promega) with Anti-Reverse Cap Analog (ARCA) or ApppG cap analogs (NEB) added in proportion 5:1 to GTP. The resulting transcripts were precipitated with 2 M LiCl. Notably, the (CAA)_5_cI-Fluc mRNA had the same coding region as the cI-Fluc construct. All mRNA transcripts were checked for integrity by denaturing urea polyacrylamide gel electrophoresis.

### Mammalian cell growth and FLERT

HEK293T cells were cultured and transferred into 24-well plates 12–16 h before transfection, in 500 μl medium per well and in an amount to finally obtain 70–80% confluency, i.e. ~10^5^ cells per well^58^. The transfection was performed using Unifectin-56 (Unifect Group, Russia). The manufacturer’s protocol was modified to obtain efficient mRNA transfection according to ref. 59. Briefly, the mixture of 0.18 μg Fluc mRNA and 0.02 μg Rluc mRNA per well, supplemented with 0.4 μl Unifectin in 140 μl DMEM, was incubated for 15 min and transferred to the cells. Stress-inducing substances were diluted with water to obtain 100^x^ solutions, as indicated, and were added to the medium (6.5 μl per well) right before the addition of the transfection complexes. All manipulations were performed in a way to minimize the time of cells holding out of CO_2_ box and to avoid cooling of the plate. Specifically, we operated in the vicinity of the CO_2_ box, with a laminar airflow being switched off. We used pre-warmed gel thermo pack for putting the plate outside the CO_2_ box and held the plate lid closed whenever possible, *i.e*. all the time except the very moments of dropping the mixtures. Right after transfection, the plate was returned to the CO_2_ box and held for the first 5 min with lid removed, in order to facilitate re-warming and CO_2_ exchange, and then the pre-warmed lid was closed. Two hours after transfection, cells were harvested, and luciferase activities were analyzed with the Dual Luciferase Assay kit (Promega). All the transfections were repeated at least three times in different cell passages. The mean values ± SD were calculated.

### Mammalian and yeast cell-free systems and *in vitro* translation assays

Krebs-2 ascite cells S30 extract was prepared as described previously^24^. Yeast cell-free extract was prepared according to^60^. Translation experiments in the mammalian system were performed in a total volume of 10 μl, which contained 5 μl of the S30 extract, translation buffer (20 mM Hepes-KOH pH 7.6, 1 mM DTT, 0.5 mM spermidine-HCl, 0.8 mM Mg(OAc)_2_, 8 mM creatine phosphate, 1 mM ATP, 0.2 mM GTP, 120 mM KOAc and 25 μM of each amino acid), 2U of RiboLock RNase inhibitor (Thermo Scientific), 0.5 mM D-luciferin (Promega), 100 ng mRNA and 1 μl of either drugs (as water solutions), proteins (in Buffer A: 20 mM Tris-HCl pH 7.5, 100 mM KCl, 10% glycerol, 1 mM DTT and 0.5 mM PMSF), or the corresponding vehicles (water or Buffer A), as indicated. Translation reactions in the yeast system were performed in a total volume of 10 μl, containing 5 μl of the extract, translation buffer (10 mM Hepes-KOH pH 7.4, 1 mM DTT, 2 mM Mg(OAc)_2_, 12 mM creatine phosphate, 1 mM ATP, 0.4 mM GTP, 75 mM KOAc and 50 μM of each amino acid), 3U of RiboLock RNase inhibitor, 0.5 mM D-luciferin, 50 ng mRNA and 1 μl of e/aIF2 dilutions in Buffer A, as indicated. Translation mixtures were incubated in a white 384-well plate (F-bottom, non-binding polystyrol, Grenier GR-781904), covered by a PCR plate seal, at 30 °C (for the mammalian system) or 25 °C (for the yeast one) in the TECAN Infinite F200 Pro plate reader with continuous measurement of the luciferase activity (integration time 3 s). Light intensities at 60 min or 30 min (for the mammalian or the yeast systems, respectively) were taken as luciferase activity values.

### Purification of translation initiation components, assembly and analysis of ribosomal complexes

eIF2, eIF3, eIF4F, eIF5B, 40 S and 60 S were purified from HeLa cell extract, eIF1, eIF1A, eIF4A, eIF4B and eIF5 were expressed in *E. coli* as described^23^,^44^,^61^. Purified tRNA_f_^Met^, a kind gift from V. Makhno and Y. Semenkov, was used as initiator tRNA. For aminoacylation, recombinant MetRSase was used as described^23^. The genes coding the α and β subunits of *S. cerevisiae* eIF2 were cloned between the NdeI and BamHI restriction sites of pET11c, expressed in *E. coli* strain BL21(DE3)/pLacIRARE and the proteins were purified using chromatography on Q-sepharose (eIF2α) or Butyl-Toyopearl 650 S (eIF2β) with the following re-chromatography on the same column. Chimeric heterotrimer of eIF2 α and β subunits with aIF2γ from *Sulfolobus solfataricus* was reconstructed as described in ref. 62.

48 S translation initiation complexes were assembled and analyzed by toe-printing assay as described earlier^23^. Briefly, 48 S complexes were assembled by incubating 0.5 pmol of mRNA for 10 min at 30 °C in a 20-μl reaction volume that contained the reconstitution buffer (20 mM Tris-HCl, pH 7.5; 110 mM KOAc; 1 mM Mg(OAc)_2_; 0.25 mM spermidine-HCl; 1 mM DTT), 0.4 mM GTP·Mg and 1 mM ATP·Mg, 10 pmol of Met-tRNA_f_^Met^, 2.5 pmol of 40 S ribosomal subunits and combination of factors (eIF1 (10 pmol), eIF1A (10 pmol), eIF2 (5 pmol), eIF3 (5 pmol), eIF4A (10 pmol), eIF4B (5 pmol), eIF4F (2 pmol), and eIF5B (5 pmol)), as described in the text. For toe-printing, [^32^P]-labeled oligonucleotide CCAGGGTTTTCCCAGTCACG was used. Primer extension analysis was performed essentially as described^61^. Images were obtained with the Typhoon FLA 9500 Phosphorimager at the Moscow State University Development Program PNR5 Centre.

## Additional Information

**How to cite this article**: Akulich, K. A. *et al*. Four translation initiation pathways employed by the leaderless mRNA in eukaryotes. *Sci. Rep.* **6**, 37905; doi: 10.1038/srep37905 (2016).

**Publisher's note:** Springer Nature remains neutral with regard to jurisdictional claims in published maps and institutional affiliations.

## Supplementary Material

Supplementary Figures
