# Supplementary material for: Four translation initiation pathways employed by the leaderless mRNA in eukaryotes
Source: Sci Rep. 2016 Nov 28;6:37905. doi: 10.1038/srep37905 (PMC5124965; doi:10.1038/srep37905)
Supplement: Supplementary Figures [file srep37905-s1.pdf]

Four translation initiation pathways employed by the leaderless mRNA in eukaryotes

Kseniya A. Akulich, Dmitry E. Andreev, Ilya M. Terenin, Victoria V. Smirnova, Aleksandra S. Anisimova, Desislava S. Makeeva, Valentina I. Arkhipova, Elena A. Stolboushkina, Maria B. Garber, Maria M. Prokofjeva, Pavel V. Spirin, Vladimir S. Prassolov, Ivan N. Shatsky, and Sergey E. Dmitriev

SUPPLEMENTARY FIGURES

**cIlacZ**	TAATACGACTCACTATA	..........g**atgagcacaaaaaagaaaccattaacacaagagcagcttCCGGATCCCGTCGTTTTACAACGT**
**cI-Fluc**	TAATACGACTCACTATA	..........g**atgagcacaaaaaagaaaccattaacacaagtgcagcttGAGGATCCCACGGAAGACGCCAAA**
**cIstop-Fluc**	TAATACGACTCACTATA	..........g**taaagcacaaaaaagaaaccattaacacaagtgcagcttGAGGATCCCACGGAAGACGCCAAA**
**(CAA)$_5$cI-Fluc**	TAATACGACTCACTATA	......GACAACAACAACAACAAGAA**atgagcacaaaaaagaaaccattaacacaagtgcagcttGAGGATCCCACGGAAGACGCCAAA**
**Actin-Fluc**	TAATACGACTCACTATA	.GGACCGCCGAGACCGCGTCCGCCCCGCGAGCACAGAGCCTCGCCTTTGCCGATCCGCCGCCCGTCCACACCCGCCGCCAGCTCACC**ATGGAAGACGCCAAA**
**Actin-Rluc**	TAATACGACTCACTATA	.GGACCGCCGAGACCGCGTCCGCCCCGCGAGCACAGAGCCTCGCCTTTGCCGATCCGCCGCCCGTCCACACCCGCCGCCAGCTCACC**ATGGCTTCGAAAGTT**

Supplementary Figure 1. **Partial nucleotide sequences of plasmids used in this study**. T7 promoters are outlined, coding regions are shown in **bold**, *Fluc* and *Rluc* sequences are yellow and blue, respectively. The cI sequence is shown in lower case.

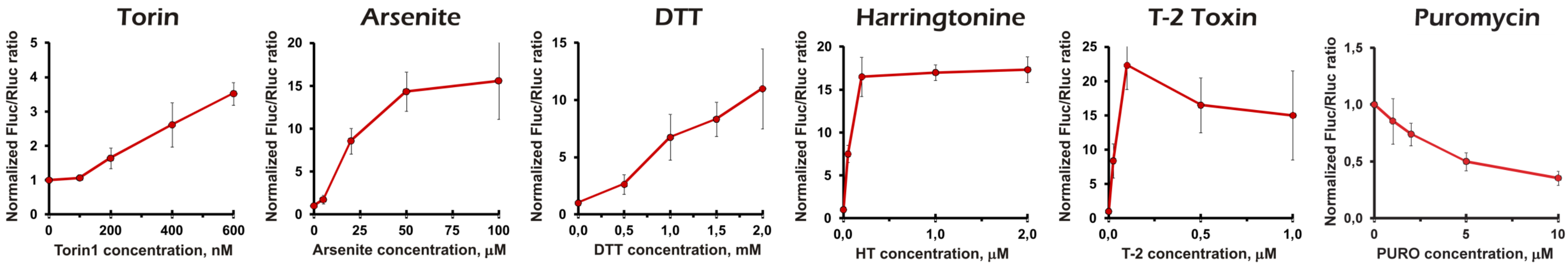

Supplementary Figure 2. **Stimulation of the leaderless cI-Fluc mRNA translation as related to that of Actin-Rluc under conditions of cell stress**. The Fluc activity units (shown in Figures 1d and 2b) were divided to that of Rluc in the same experiments and then normalized to the values calculated for the wells without drugs.

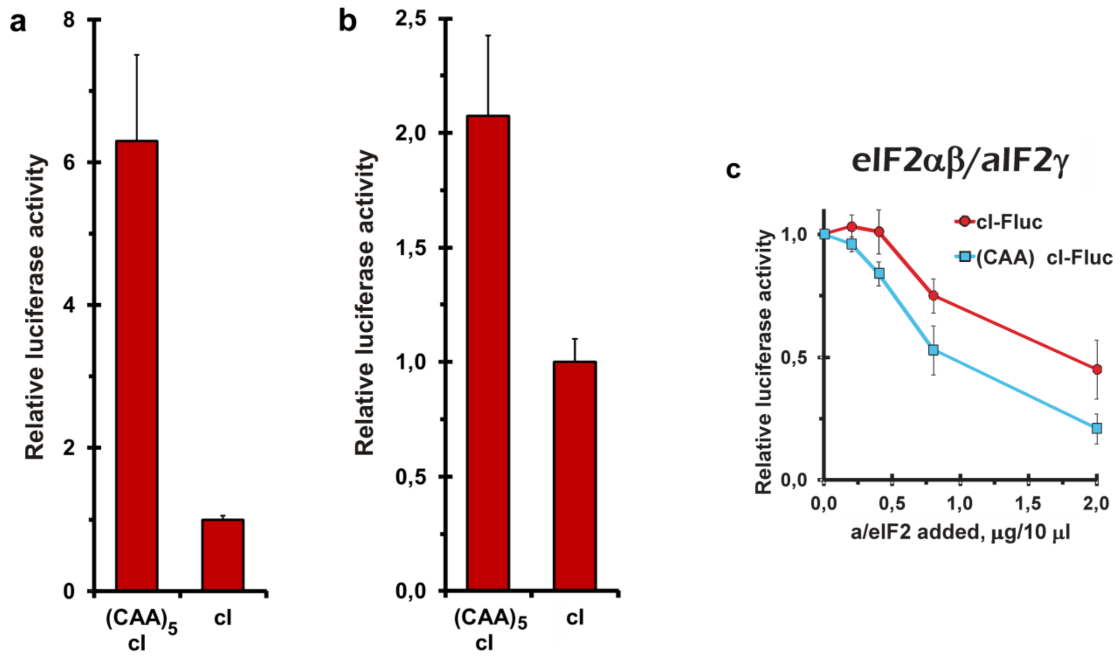

Supplementary Figure 3. Translational properties of the leaderless cI-Fluc and the leadered $(CAA)_5$cI-Fluc mRNAs in cell-free systems. (a) Translation efficiency of the two mRNAs in mammalian cell-free system prepared from Krebs-2 cells extract. Luciferase activity values were normalized to that produced by the cI-Fluc mRNA. (b) Translation of the same mRNAs in yeast cell-free system. (c) Effects of recombinant yeIF2αβ/aIF2γ chimeric heterotrimer on the mRNA translation levels in the yeast cell-free system. The values obtained in experiment, where buffer was added instead of the protein, were taken as 1 for each of the mRNAs.
